# Preferences for Health Information Technologies Among US Adults: Analysis of the Health Information National Trends Survey

**DOI:** 10.2196/jmir.9436

**Published:** 2018-10-18

**Authors:** Onur Asan, Farion Cooper II, Sneha Nagavally, Rebekah J Walker, Joni S Williams, Mukoso N Ozieh, Leonard E Egede

**Affiliations:** 1 School of Systems and Enterprises Stevens Institute of Technology Hoboken, NJ United States; 2 Center for Advancing Population Science (CAPS) Medical College of Wisconsin Milwaukee, WI United States

**Keywords:** chronic conditions, eHealth, health information exchange, health information technology, internet, mHealth, mobile phone

## Abstract

**Background:**

Emerging health technologies are increasingly being used in health care for communication, data collection, patient monitoring, education, and facilitating adherence to chronic disease management. However, there is a lack of studies on differences in the preference for using information exchange technologies between patients with chronic and nonchronic diseases and factors affecting these differences.

**Objective:**

The purpose of this paper is to understand the preferences and use of information technology for information exchange among a nationally representative sample of adults with and without 3 chronic disease conditions (ie, cardiovascular disease [CVD], diabetes, and hypertension) and to assess whether these preferences differ according to varying demographic variables.

**Methods:**

We utilized data from the 2012 and 2014 iteration of the Health Information National Trends Survey (N=7307). We used multiple logistic regressions, adjusting for relevant demographic covariates, to identify the independent factors associated with lower odds of using health information technology (HIT), thus, identifying targets for awareness. Analyses were weighted for the US population and adjusted for the sociodemographic variables of age, gender, race, and US census region.

**Results:**

Of 7307 participants, 3529 reported CVD, diabetes, or hypertension. In the unadjusted models, individuals with diabetes, CVD, or hypertension were more likely to report using email to exchange medical information with their provider and less likely to not use any of the technology in health information exchange, as well as more likely to say it was not important for them to access personal medical information electronically. In the unadjusted model, additional significant odds ratio (OR) values were observed. However, after adjustment, most relationships regarding the use and interest in exchanging information with the provider were no longer significant. In the adjusted model, individuals with CVD, diabetes, or hypertension were more likely to access Web-based personal health information through a website or app. Furthermore, we assessed adjusted ORs for demographic variables. Those aged >65 years and Hispanic people were more likely to report no use of email to exchange medical information with their provider. Minorities (Hispanic, non-Hispanic black, and Asian people) were less likely to indicate they had no interest in exchanging general health tips with a provider electronically.

**Conclusions:**

The analysis did not show any significant association among those with comorbidities and their proclivity toward health information, possibly implying that HIT-related interventions, particularly design of information technologies, should focus more on demographic factors, including race, age, and region, than on comorbidities or chronic disease status to increase the likelihood of use. Future research is needed to understand and explore more patient-friendly use and design of information technologies, which can be utilized by diverse age, race, and education or health literacy groups efficiently to further bridge the patient-provider communication gap.

## Introduction

The burden of chronic diseases continues to grow, and they remain the most common cause of death and disability worldwide [1]. More than half of the adults in the United States have at least one chronic condition (eg, hypertension, coronary heart disease, stroke, diabetes, and cancer) [2,3]. Cardiovascular disease (CVD) and diabetes are two common chronic conditions that share common risk factors including age, genetic factors, obesity, poor nutrition, impaired glucose tolerance, and physical inactivity [4]. Moreover, diabetes is a well-known risk factor for CVD as it can increase atherosclerosis and cause elevated blood pressure and cholesterol levels in many individuals [4]. In addition, hypertension is a well-established risk factor for CVD, and studies have shown a relationship between hypertension and both vascular and structural cardiac remodeling [5]. Furthermore, inflammatory mediators such as damage-associated molecular patterns have been associated with CVD, hypertension, and diabetes [6]. Immune-mediated and adaptive immune responses have been implicated in hypertension and other vascular diseases as well [7]. Death from CVD rose by >40% between 1990 and 2013 as a result of factors such as population increase, aging, and epidemiological changes in chronic diseases [8]. Thus, it is critical for the population with chronic diseases to maintain effective disease management to achieve an improved quality of life and health outcomes [9,10].

The need for continuous monitoring, long-term planning, and frequent interaction of patients with chronic diseases with their providers might be addressed with ongoing technological advancements [1]. Emerging health technologies such as remote patient monitoring systems, mobile phone intervention programs, electronic health databases, smartphone apps, instant messaging, video calling, and patient portals are increasingly being used in health care for communication, data collection, patient monitoring, education, and facilitating adherence to chronic disease management [11-28]. It has been suggested that these technologies can assist in reducing both the burden and cost of CVD around the world [29-31].

Specifically, the use of Web-based and mobile health (mHealth) intervention programs has led to a few suggestions for the technological intervention to improve health information exchange (HIE). In fact, it has been shown that information exchange via various portals and mobile intervention has led to an increased adherence to healthy lifestyle programs, treatment regimens, and disease prevention programs [11-13,15,32]. However, the literature on this topic is very general and seldom concentrates on different chronic diseases and conditions. Literature that does converge on specific diseases like diabetes and chronic kidney disease only concentrate on one type of electronic health (eHealth) technology at a time [32,33]. The evidence that concentrates on the broad use of technology in addressing CVD specifically is limited [34]. There is also a lack of studies on differences in the preference for using information exchange technologies between patients with chronic and nonchronic diseases and factors affecting these differences. From a design and intervention development perspective, it would be critical to understand user differences and their relationship with demographic factors to augment the uptake of the newly developed information technologies.

The purpose of this paper is to understand the preferences and use of technology for information exchange among a nationally representative sample of adults with and without 3 chronic disease conditions (ie, CVD, diabetes, and hypertension) and to assess whether these preferences differ according to varying demographic variables.

## Methods

### Sample Population

Data for this study were derived from the Health Information National Trends Survey (HINTS). HINTS is a large-scale, household interview survey of US noninstitutionalized adults aged ≥18 years. HINTS gathers information from the general population to investigate trends in the utilization of health communication systems between providers and patients, specifically pertaining to access and usage [35]. We utilized data from HINTS 4 Cycles 2 (2012) and 4 (2014), which were obtained using the single mode mailing service and the Next Birthday Method for respondent selection. To perform a consistent selection of respondents across all households, we used the Next Birthday Method; in this technique, adults who have a next birthday coming up are requested to finish the survey for every family unit.

The sampling frame came from a collection of databases used by the Marketing Systems Group to obtain a random sample of addresses. This was then grouped into 3 specific sampling strata: (1) high concentrations of minority populations; (2) low concentrations of minority populations; and (3) counties in Central Appalachia (regardless of minority populations) [35]. A total of 12,055 households received the 4-part mailed questionnaire in Cycle 2, and 13,996 households received the 4-part mailed questionnaires in Cycle 4. The response rate was 39.97% (4818/12,055) for Cycle 2 and 34.44% (4820/13,996) for Cycle 4 [36,37]. Additional information about data collection and methodologies can be found in the corresponding methodology reports for HINTS 4 Cycles 2 and 4. The sampling methodology allowed for weighting of the sample to provide population estimates [36,37]. Further details on survey design and sampling strategies of the overall HINTS mechanism were published in a previous work [38].

We identified individuals with CVD, diabetes, or hypertension through self-reported diagnosis via the questions “Has a doctor or other health professional ever told you that you have diabetes or high blood sugar,” “Has a doctor or other health professional ever told you that you have high blood pressure or hypertension?” and “Has a doctor or other health professional ever told you that you have a heart condition such as heart attack, angina, or congestive heart failure?” Individuals included in the sample population answered yes to any of the diagnoses. *“Missing data”* or “Inapplicable” response type for these three questions was considered to be a missing value for having a chronic condition. These 2 versions of HINTS resulted in a sample of 7307 total individuals who answered relevant questions and for whom demographic data were collected, and this was the sample size used for this analysis [36,37].

### Measures

The main outcome variables were questions asking about exchanging information with providers and accessing medical records. The questions that were included in the analysis and the responses dichotomized have been detailed in Multimedia Appendix 1.

We modeled negative responses using multiple logistic regressions adjusting for relevant covariates to identify independent factors associated with lowers odds of using health information technology (HIT) and, therefore, identify targets for awareness. Analyses were weighted for the US population and adjusted for the sociodemographic variables of age, gender, race, and US census region. Covariates were categorized as age (18-34 years, 35-44 years, 45-64 years, and 65-110 years); gender (female, male); race (Hispanic, non-Hispanic white, non-Hispanic black or African American, non-Hispanic Asian, and non-Hispanic other [non-Hispanic American Indian or Alaska Native or non-Hispanic, Native Hawaiian or other Pacific Islander or non-Hispanic multiple races mentioned]); and region (Northeast, Midwest, South, and West).

### Statistical Analysis

First, basic demographic statistics were conducted using demographic attributes: age (“18-34,” “35-44,” “45-64,” “≥65” years), gender (“Male,” “Female”), race (“Hispanic,” “non-Hispanic white,” “non-Hispanic black or African American,” “non-Hispanic Asian,” “non-Hispanic other”), and census region (“Northeast,” “Midwest,” “South,” “West”). We calculated weighted population frequencies and percentiles. Second, we analyzed each question with an unadjusted logistic regression model, followed by an adjusted logistic regression model. To model for dichotomous outcome variables, we used logit model. In a logit model, log odds of a dependent variable are modeled as a linear combination of independent variables. To implement the logit model, we used generalized linear model function and linked the binomial family to logistic regression to develop a logistic model for the analyses. Models were adjusted for age, race, gender, and region. All analyses were conducted using R statistical tool, with a *P*<.05 considered statistically significant. To analyze the weighted sample, we implemented the R Survey package with type “JKn” to include the weight samples across the dataset. Furthermore, we used sample weights from the survey data to analyze weighted population estimates and replicate weights to compute SE of estimates using the jackknife replication method.

## Results

In all, 7307 participants (weighted population of 230,993,888) answered the questions used in this analysis. Within this sample, 3529 participants (weighted population of 90,748,995) reported CVD, diabetes, or hypertension. In addition, 3.33% (243/7307) of observations were with missing data. Table 1 shows the demographics of the population. Of those with CVD, diabetes, or hypertension, 42.35% (1482/3529) were aged 45-64 years and 31.06% (1094/3529) were aged ≥65 years. Furthermore, non-Hispanic white people represented 60.79% (2142/3529) of the population, and the Southern region of the United States represented 39.25% (1383/3529) of the population.

Table 2 displays the unadjusted and adjusted odds ratios (ORs) for HIT use and interest in exchanging information with a provider. In the unadjusted models, individuals with diabetes, CVD, or hypertension were more likely to report using email to exchange medical information with their provider (OR 1.431; 95% CI 1.113-1.838) and less likely to not use any of the technology in HIE (OR 0.778; 95% CI 0.618-0.979). Additionally, those with diabetes, CVD, or hypertension were less likely to report that health apps never led to asking new questions of their provider (OR 0.526; 95% CI 0.331-0.838) and were also more likely to report having no interest in exchanging electronic appointment reminders (OR 1.818; 95% CI 1.388-2.380), in exchanging general health tips with health care provider electronically (OR 1.283; 95% CI 1.004-1.639), in exchanging medication reminders with health care provider electronically (OR 1.440; 95% CI 1.135-1.827), in exchanging diagnostic information with health care provider electronically (OR 1.269; 95% CI 1.011-1.592), or in exchanging symptoms with health care provider electronically (OR 1.263; 95% CI 1.004-1.588). However, after adjustment, all relationships regarding the use of and interest in exchanging information with the provider were no longer significant.

Table 3 displays the unadjusted and adjusted ORs for interest in accessing personal medical information. In the unadjusted models, individuals with CVD, diabetes, or hypertension were more likely to say that it was not important for them to access personal medical information electronically (OR 1.496; 95% CI 1.142-1.959); however, in the adjusted models, these individuals were more likely to access Web-based personal health information through a website or app (OR 1.877; 95% CI 1.210-2.912).

Because a number of relationships lost significance after adjustment for demographic covariates, Multimedia Appendix 2 displays the adjusted ORs for demographic variables included in models presented in Tables 2 and 3 that showed significant unadjusted differences by chronic disease diagnosis to determine which demographic variables explained the relationship. Models in Multimedia Appendix 2 were adjusted by demographic covariates age, gender, race or ethnicity, and census region and the primary variable (presence of diabetes, CVD, or hypertension). Individuals aged >65 years (OR 2.32; 95% CI 1.55-3.49) and Hispanic people (OR 1.95; 95% CI 1.26-3.01) were more likely to report no use of email to exchange medical information with their provider. Conversely, non-Hispanic black people were less likely to report that they never used an app that led to questions for their provider (OR 0.32; 95% CI 0.16-0.63), whereas individuals living in the Western region of the country were more likely to report never using an app that led to questions or a second opinion (OR 2.18; 95% CI 1.08-4.41). Minorities were less likely to indicate that they had no interest in exchanging general health tips with a provider electronically (Hispanic people [OR 0.55; 95% CI 0.34-0.88], non-Hispanic black people [OR 0.36; 95% CI 0.23-0.56], and Asian people [OR 0.33; 95% CI 0.16-0.70]). In addition, non-Hispanic black people were less likely to report having no interest in exchanging medication reminders with providers (OR 0.62; 95% CI 0.41-0.95). Individuals aged 45-64 and >65 years were more likely to report no interest in exchanging a variety of medical information with their providers and more likely to say that it was not important to access their own information electronically. However, individuals aged >65 years were less likely to say that they never accessed personal medical information electronically in the past 12 months (OR 0.25; 95% CI 0.13-0.47). Finally, women were more likely to report no interest in exchanging diagnostic information with their provider (OR 1.32; 95% CI 1.03-1.71).

**Table 1:** Weighted population demographic characteristics: sample n=7307, weighted population N=230,993,888.

Demographics		Weighted population, n (%)		*P* value
		Population with diabetes, CVD^a^, or hypertension (n=3529)	Population without diabetes, CVD, or hypertension (n=3535)	
**Gender**				.59
	Male	1683 (47.76)	1672 (47.34)	
	Female	1845 (50.19)	1863 (51.53)	
**Age (years)**				<.001^b^
	18-34	351 (9.83)	1512 (42.80)	
	35-44	458 (13.19)	671 (19.02)	
	45-64	1482 (42.35)	996 (28.19)	
	>65	1094 (31.06)	267 (7.63)	
**Race**				.12
	Hispanic	413 (11.74)	530 (14.98)	
	Non-Hispanic white	2142 (60.79)	2177 (61.63)	
	Non-Hispanic black or African American	399 (11.35)	335 (9.48)	
	Non-Hispanic Asian	92 (2.61)	198 (5.62)	
	Non-Hispanic other	81 (2.34)	70 (1.96)	
**Region**				.22
	Northeast	620 (17.58)	664 (18.86)	
	Midwest	780 (22.16)	752 (21.28)	
	South	1383 (39.25)	1260 (35.65)	
	West	741 (21.00)	856 (24.22)	

^a^CVD: cardiovascular disease.

^b^Statistically significant relationships at *P*<.05, with the presence of diabetes, cardiovascular disease, or hypertension being the primary indicator.

**Table 2:** Unadjusted and adjusted odds ratios (ORs) for the use of and interest in exchanging information with a provider among individuals with the presence of a chronic condition.

Odds^a^			Unadjusted OR (95% CI)		Adjusted OR (95% CI)	
**Odds of reporting use**						
	**In the past 12 months, used any of following to exchange info with health care professional?**					
		Email		1.431 (1.113-1.838)^b^		1.128 (0.865-1.470)
		Short message service text message		1.039 (0.688-1.571)		0.955 (0.594-1.536)
		App		0.983 (0.602-1.604)		0.734 (0.426-1.262)
		Video		1.922 (0.560-6.599)		1.379 (0.310-6.134)
		Social media		0.936 (0.499-1.756)		0.659 (0.325-1.337)
		Fax		0.874 (0.553-1.379)		0.811 (0.503-1.308)
		None		0.778 (0.618-0.979)^b^		0.963 (0.753-1.233)
**Odds of responding "no" or "never"**						
	Have apps on smartphone or tablet related to health led to asking doctor new questions or getting second opinion from another doctor?			0.526 (0.331-0.838)^b^		0.591 (0.341-1.022)
	How interested in exchanging appointment reminders with health care provider electronically?			1.818 (1.388-2.380)^b^		1.072 (0.723-1.588)
	How interested in exchanging general health tips with health care provider electronically?			1.283 (1.004-1.639)^b^		1.037 (0.770-1.397)
	How interested in exchanging medication reminders with health care provider electronically?			1.440 (1.135-1.827)^b^		0.975 (0.718-1.324)
	How interested in exchanging lab or test results with health care provider electronically?			1.215 (0.951-1.552)		0.938 (0.683-1.289)
	How interested in exchanging diagnostic information with health care provider electronically?			1.269 (1.011-1.592)^b^		0.996 (0.757-1.312)
	How interested in exchanging vital signs with health care provider electronically?			1.259 (0.986-1.607)		1.022 (0.754-1.386)
	How interested in exchanging lifestyle behaviors with health care provider electronically?			1.187 (0.942-1.496)		0.882 (0.669-1.163)
	How interested in exchanging symptoms with health care provider electronically?			1.263 (1.004-1.588)^b^		0.832 (0.621-1.116)

^a^The model was adjusted by demographic covariates: age, gender, race or ethnicity, and census region.

^b^Statistically significant relationships at *P*<.05, with the presence of diabetes, cardiovascular disease, or hypertension being the primary indicator.

**Table 3:** Unadjusted and adjusted odds ratios (ORs) for use of and interest in accessing personal medical information among individuals with the presence of a chronic condition.

Odds^a^		Unadjusted OR (95% CI)		Adjusted OR (95% CI)	
**Odds of responding not important or not confident or no or more times**					
	How important is it for doctors to share your medical information with each other electronically?		0.960 (0.680-1.355)		1.282 (0.790-2.080)
	How important would it be for you to access personal medical information electronically?		1.496 (1.142-1.959)^b^		1.089 (0.761-1.557)
	How confident are you that safeguards are present to protect your medical records from being seen by people who are not permitted to view them?		0.817 (0.638-1.048)		0.902 (0.670-1.213)
	Have you been offered access to personal health info through a website or app by your health care provider?		1.043 (0.837-1.301)		1.041 (0.817-1.324)
**Odds of increasing number of times**					
	In the past 12 months, how many times did you access personal health information through a website or app?		1.251 (0.824-1.898)		1.877 (1.210-2.912)^b^

^a^The model was adjusted by demographic covariates: age, gender, race or ethnicity, and census region.

^b^Statistically significant relationships at *P*<.05, with the presence of diabetes, cardiovascular disease, or hypertension being the primary indicator.

## Discussion

### Principal Findings

In this study, we examined and compared patients’ attitudes toward the use of health information technologies for HIE between providers and patients with and without the chronic (diabetes, CVD, or hypertension) conditions. The analyses showed that while unadjusted differences existed in responses to the use of HIT based on the existence of chronic disease, the inclusion of the demographic factors of gender, race, age, and region of residence removed this statistical significance and may explain the differences. This suggests that demographic differences are more important than differences due to presence or absence of a chronic disease. This is particularly important as interventions are developed to increase the use of HIT across different population groups in the United States. Specifically, our findings suggest that future interventions should target the unique needs of the elderly and ethnic minorities regardless of their disease status.

Our findings show that older individuals, regardless of the presence of a chronic condition, indicated less interest in using HIT to communicate or exchange information with their provider. Previous studies have also reported similar findings. One study showed the rate of HIT use among those aged ≥65 years to be less than that among younger patients [39]. Another study also showed that older adults were less likely than younger adults to value the importance of Patient Health Records [33]. There might be several reasons behind these findings, and they include the following: (1) greater ease of use and comfort with technology among younger adults [40]; (2) poor usability, availability, and accessibility of HIT functions tailored to the elderly [41-43]; (3) lower health literacy in the elderly [44,45]; (4) lower educational attainment and income in the elderly [3,46]; and (5) unique challenges from having chronic diseases that are more prevalent in the elderly and that impede the use of technology (eg, arthritis, vision impairment) [47]. However, the potential of technology for better communication and disease management is clear [48]; therefore, given the burden of chronic disease in the elderly, it is imperative to develop more user-friendly interfaces to facilitate better use of HIT for this age group. In addition, the results showed that adults aged 65 years with chronic disease reported no interest in exchanging medical information through the Web with their doctors, although they stated that they accessed health info electronically in the past 12 months. This might show that this group would like to access information on the Web to get informed, but still prefer other type of communication channels to discuss with their providers, such as in person or via phone over electronic information exchange. Finally, a recent study has shown increased use of mHealth technologies by the elderly group [49]; likewise, this study also signals that if these technologies are designed to be more patient friendly and if they address cognitive load as well as understand the needs of elderly patients, we would witness increased use and more eHealth information exchange among this group too.

In addition, we found that minorities showed more interest in using HIT, particularly for general health tips and medication reminders. In addition, non-Hispanic black people were less likely to report that they never used an app that led to questions for their provider. Some previous studies have shown that minorities are less likely to seek out Web-based health information [48], but our results show that this trend may be changing given the advancement in technology and rate of possession of mobile phones among minority populations. A recent Pew Internet & American Life Project’s 2013 Health Online study has also shown that minorities (Hispanic and non-Hispanic black people) reported using their mobile phones to access health information, especially information related to pregnancy and weight, more than non-Hispanic white people [50,51]. It is also interesting to see that given the increased use of smartphone among minorities to access health information through app or Web, they are not more likely to report or exchange information with their providers. This changing trend suggests that future interventions for ethnic minorities should take greater advantage of technology, especially eHealth and mobile technology, which can also address and solve some disparity problems in the long run.

Some past studies have shown mixed results on gender differences in the eHealth use and perception of HIE [52-54]. In our study, the only question indicating a difference by gender suggests that men may be more interested in using HIT to discuss diagnostic information with their providers. Finally, individuals with chronic conditions were more likely to access Web-based personal health information through a website or app for the last 12 months. One reason for this finding might be that individuals with no chronic condition did not need or have any issue requiring them to access their health records compared with individuals with chronic conditions.

### Limitations

In this analysis, we used a large, population-based sample providing generalizable results and investigated a variety of HIT options; however, there are some limitations worth noting. First, the data were cross-sectional and, therefore, cannot offer information on causality. Second, the response rate for HINTS was 21%-35%, and therefore, it may have more selection bias than other national surveys. Local or regional studies that provide more detailed data with a higher response rate should be conducted to validate these findings. Finally, there are several possible confounders that may further explain the relationships noted that were not included in the dataset.

### Conclusions

This study compares the HIT use for information exchange by individuals with and without chronic conditions (diabetes, CVD, or hypertension) and potential factors that influence HIT use. The findings did not show any significant association among those with comorbidities and their proclivity toward health information. This study suggests that HIT-related interventions, particularly the design of mHealth technologies, should focus more on demographic factors, including race, age, and region, than on chronic disease status or comorbidities to increase the likelihood of use. Future research is needed to understand and explore more patient-friendly use and design of mHealth apps, which can be utilized by various age, race, and education or health literacy groups efficiently to further bridge the communication gap between patients and their provider. A more qualitative exploration would be beneficial to identify why certain groups do and do not use HIT for HIE purposes.

## Appendix

Multimedia Appendix 1 Survey questions used in the analysis.

Multimedia Appendix 2 Adjusted odds ratios for selected questions with demographics information.

## Abbreviations

CVD: Cardiovascular disease
eHealth: electronic health
HIE: health information exchange
HINTS: Health Information National Trends Survey
HIT: Health Information Technology
mHealth: mobile health
OR: odds ratio
