# Supplementary material for: Preferences for Health Information Technologies Among US Adults: Analysis of the Health Information National Trends Survey
Source: J Med Internet Res. 2018 Oct 18;20(10):e277. doi: 10.2196/jmir.9436 (PMC6245956; doi:10.2196/jmir.9436)
Supplement: Multimedia Appendix 1 [file jmir_v20i10e277_app1.pdf]

1) In the past 12 months, have you used any of the following to exchange medical information with a health care professional? With response options of ‘E-mail only’, ‘Text message only’, ‘App on a smart phone or mobile device only’, ‘Video conference (e.g., Skype, Facetime, etc.) only’, ‘Social media (e.g., Facebook, Google+, Caring Bridge, etc.) only’, ‘Fax only’, ‘None’. (None vs. Other responses)
2) Have the apps on your smartphone or tablet related to health led you to ask a doctor new questions, or to get a second opinion from another doctor? With response options of ‘Yes’, ‘No’. (No vs. Yes)

Questions 3 to 11 examined interest of people regarding information exchange electronically and used the response options of “Very”, “Somewhat”, A little”, “Not at all” Responses were dichotomized as (Not at all vs. very/somewhat/a little).

3) How interested are you in exchanging appointment reminders with a health care provider electronically?
4) How interested are you in exchanging general health tips with a health care provider electronically?
5) How interested are you in exchanging medication reminders with a health care provider electronically?
6) How interested are you in exchanging lab/test results with a health care provider electronically?
7) How interested are you in exchanging diagnostic information (e.g., medical illnesses or diseases) with a health care provider electronically?
8) How interested are you in exchanging vital signs (e.g., heart rate, blood pressure, glucose levels, etc.) with a health care provider electronically?
9) How interested are you in exchanging lifestyle behaviors (e.g., physical activity, food intake, sleep patterns, etc.) with a health care provider electronically?
10) How interested are you in exchanging symptoms (e.g., nausea, pain, dizziness, etc.) with a health care provider electronically?

Questions 11-13 examined other aspects of technology use and have the response options of “Very important”, “Somewhat important”, or “Not at all important” Responses were dichotomized as (Not at all important vs. very important/somewhat important).

11) Please indicate how important each of the following statements is to you. Doctors and other health care providers should be able to share your medical information with each other electronically?
12) How important would it be for you to get your own medical information electronically?
13) How confident are you that safeguards (including the use of technology) are in place to protect your medical records from being seen by people who aren't permitted to see them?
14) Have you ever been offered access to your own personal health information online through a secure website or app by your health care provider? With response options of ‘Yes’, ‘No’. (Dichotomized as No vs. Yes)
15) How many times did you access your personal health information on-line through a secure website or app in the last 12 months? With response options of ‘None’, ‘1 to 2 times’, ‘3 to 5 times’, ‘6 to 9 times’, ‘10 or more times’. (Dichotomized as None vs. 1-2/3-5/6-9/10 or more times)
