# Supplementary material for: Preferences for Health Information Technologies Among US Adults: Analysis of the Health Information National Trends Survey
Source: J Med Internet Res. 2018 Oct 18;20(10):e277. doi: 10.2196/jmir.9436 (PMC6245956; doi:10.2196/jmir.9436)
Supplement: Multimedia Appendix 2 [file jmir_v20i10e277_app2.pdf]

Multimedia Appendix 2. Adjusted odds ratios for selected questions with demographics information.

	Odds of reporting use		Odds of Reporting No or Never							
	Use of e-mail	No exchange of info	App use for questions	Interest in Appointment reminders	Interest in General Health Tips	Interest in Medication Reminders	Interest in Diagnostic Info	Interest in Symptoms	Important to Access info	Accessed own info
**Gender**										
Male	Reference									
Female	1.04 (0.78 - 1.37)	0.97 (0.75 - 1.26)	0.81 (0.47 - 1.41)	1.08 (0.79 - 1.46)	1.21 (0.92 - 1.60)	1.10 (0.84 - 1.45)	**1.32 (1.03 - 1.71)**	1.07 (0.82 - 1.38)	1.06 (0.79 - 1.43)	1.12 (0.70 - 1.80)
**Age**										
18-34	Reference									
35-44	1.35 (0.88 - 2.06)	0.89 (0.60 - 1.33)	0.85 (0.39 - 1.85)	1.84 (0.87 - 3.88)	1.00 (0.61 - 1.65)	1.19 (0.69 - 2.04)	1.14 (0.73 - 1.78)	1.09 (0.67 - 1.79)	1.23 (0.66 - 2.31)	0.51 (0.25 - 1.04)
45-64	1.33 (0.93 - 1.91)	0.91 (0.65 - 1.28)	0.86 (0.46 - 1.60)	**3.27 (1.66 - 6.45)**	1.21 (0.79 - 1.84)	**2.24 (1.39 - 3.60)**	**1.47 (1.00 - 2.16)**	**1.89 (1.24 - 2.87)**	**1.77 (1.01 - 3.09)**	0.68 (0.39 - 1.16)
above 65	**2.32 (1.55 - 3.49)**	0.45 (0.31 - 0.66)	0.38 (0.17 - 0.86)	**6.22 (3.02 - 12.81)**	**1.92 (1.22 - 3.04)**	**3.82 (2.28 - 6.40)**	**2.29 (1.50 - 3.49)**	**3.50 (2.21 - 5.53)**	**4.84 (2.73 - 8.59)**	**0.25 (0.13 - 0.47)**
**Race**										
NHW	Reference									
Hispanic	**1.95 (1.26 - 3.01)**	0.78 (0.53 - 1.14)	0.55 (0.28 - 1.07)	0.95 (0.59 - 1.53)	**0.55 (0.34 - 0.88)**	0.66 (0.43 - 1.01)	1.05 (0.72 - 1.53)	1.02 (0.67 - 1.54)	1.27 (0.80 - 2.03)	0.20 (0.09 - 0.44)
NHB	1.27 (0.84 - 1.93)	0.96 (0.65 - 1.40)	**0.32 (0.16 - 0.63)**	0.85 (0.49 - 1.49)	**0.36 (0.23 - 0.56)**	**0.62 (0.41 - 0.95)**	1.04 (0.70 - 1.55)	0.81 (0.54 - 1.21)	0.79 (0.49 - 1.28)	0.61 (0.33 - 1.14)

Asian	1.22 (0.60 - 2.48)	1.24 (0.66 - 2.36)	1.38 (0.46 - 4.17)	1.04 (0.52 - 2.08)	**0.33 (0.16 - 0.70)**	0.76 (0.40 - 1.47)	0.58 (0.29 - 1.13)	0.63 (0.32 - 1.23)	1.77 (0.87 - 3.59)	0.78 (0.24 - 2.55)
Other	**0.43 (0.21 - 0.89)**	**2.62 (1.31 - 5.24)**	0.47 (0.13 - 1.70)	1.04 (0.34 - 3.19)	0.80 (0.35 - 1.82)	0.74 (0.33 - 1.69)	1.24 (0.60 - 2.58)	0.79 (0.35 - 1.77)	0.89 (0.39 - 2.04)	0.91 (0.28 - 3.01)
**Region**										
Northeast	Reference									
Midwest	0.90 (0.57 - 1.42)	1.10 (0.73 - 1.67)	0.99 (0.45 - 2.15)	1.03 (0.62 - 1.71)	0.98 (0.62 - 1.52)	0.91 (0.59 - 1.40)	1.02 (0.70 - 1.49)	0.87 (0.58 - 1.31)	0.76 (0.48 - 1.21)	0.57 (0.27 - 1.20)
South	1.11 (0.74 - 1.66)	0.89 (0.62 - 1.29)	1.43 (0.71 - 2.86)	0.81 (0.51 - 1.29)	1.06 (0.71 - 1.58)	0.89 (0.59 - 1.33)	1.05 (0.74 - 1.48)	0.87 (0.60 - 1.25)	0.93 (0.61 - 1.43)	0.59 (0.31 - 1.15)
West	**0.58 (0.38 - 0.89)**	1.45 (0.97 - 2.16)	**2.18 (1.08 - 4.41)**	0.66 (0.41 - 1.07)	0.85 (0.54 - 1.34)	0.69 (0.43 - 1.09)	0.81 (0.53 - 1.23)	**0.57 (0.36 - 0.89)**	0.80 (0.49 - 1.31)	0.97 (0.49 - 1.90)

Note: bold indicates relationships statistically significant at P<0.005. Model adjusted by demographic covariates: Age, Gender, Race/Ethnicity and Census Region, and the primary variable presence of diabetes/CVD/hypertension.
